# Treatment of chronic telogen effluvium with oral minoxidil: A retrospective study

**DOI:** 10.12688/f1000research.11775.1

**Published:** 2017-09-06

**Authors:** Eshini Perera, Rodney Sinclair

**Affiliations:** 1 Sinclair Dermatology, East Melbourne, VIC, Australia; 2 Department of Medicine, University of Melbourne, Parkville, VIC, Australia

**Keywords:** androgenic, androgenetic, alopecia, hair loss, shedding, baldness

## Abstract

**Background**: Chronic telogen effluvium (CTE) may be primary or secondary to various causes, including drug reaction, nutritional deficiency and female pattern hair loss (FPHL).  Oral minoxidil stimulates hair growth, and topical minoxidil is used in the treatment of FPHL and male androgenetic alopecia. minoxidil has not been used to treat CTE. This study aimed to assess the treatment of CTE with once daily oral minoxidil.

**Methods**: Women with a diagnosis of CTE based on >6 month history of increased telogen hair shedding, no visible mid frontal scalp hair loss (Sinclair stage 1) and no hair follicle miniaturization on scalp biopsy were treated with once daily oral minoxidil.  Hair shedding scores (HSS) at baseline, 6 and 12 months were analysed using the Wilcoxon rank sum test for pair-wise comparisons.

**Results**: Thirty-six women were treated with oral minoxidil (range, 0.25-2.5 mg) daily for 6 months.  Mean age was 46.9 years (range 20-83), HSS at baseline was 5.64, and duration of diagnosis was 6.55 years (range 1-27).  There was a reduction in mean HSS scores from baseline to 6 months of 1.7 (p<0.001) and baseline to 12 months of 2.58 (p<0.001). Five women who described trichodynia at baseline, noted improvement or resolution within 3 months.  Mean change in blood pressure was minus 0.5 mmHg systolic and plus 2.1 mmHg diastolic.  Two patients developed transient postural dizziness that resolved with continued treatment.  One patient developed ankle oedema.  Thirteen women developed facial hypertrichosis.  For 6 patients this was mild and did not require treatment; 4 had waxing of their upper lip or forehead; 3 had laser hair removal.  No patients developed any haematological abnormality.  All 36 women completed 12 months of treatment.

**Conclusions**: Once daily oral minoxidil appears to reduce hair shedding in CTE.  Placebo controlled studies are recommended to further assess this response.

## Introduction

**Figure 1.**
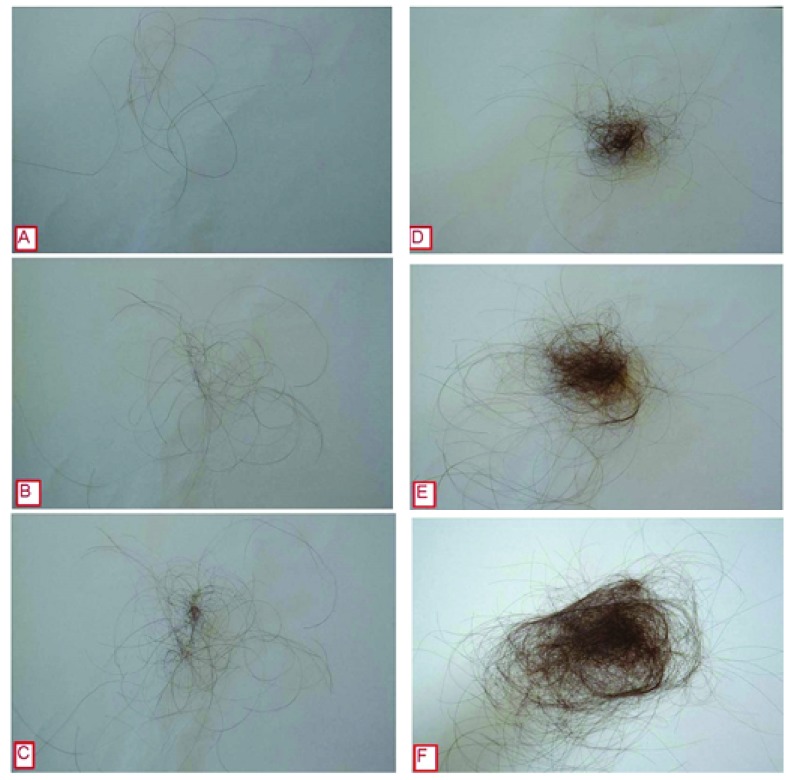
Hair shedding scale. Scores vary between 1 (minimal shedding) to 6 (copious hair shedding) ^12^.

Hair shedding severity can be scored using a visual analogue scale ^1^. For women with long hair, a shedding severity of 1, 2 or 3 is considered normal; severity 4 is borderline; while a shedding severity of 5 or 6 is excessive ( Figure 1). Visual inspection of shed hair bulbs will determine if the hairs shed are being lost during the anagen phase of the hair cycle or the telogen phase. This examination of the bulb provides an important clue to the aetiology of the hair loss ^2^.

Telogen effluvium is a non-scarring alopecia characterised by excessive shedding of telogen club hair diffusely from the scalp. It generally begins 8–12 weeks after a triggering event, such as pregnancy, major illness or complicated surgery, and resolves within 3–6 months. Once resolved, self-limiting telogen effluvium can be retrospectively diagnosed as acute telogen effluvium ^3^. Telogen shedding that persists beyond 6 months is called chronic telogen effluvium (CTE) ^4^. CTE may be primary or secondary to a range of triggers, including androgenetic alopecia (AGA), nutritional deficiency, endocrinopathy, connective tissue disease or drug induced ^5^. The aetiology of primary CTE is unknown ^4^. Mathematical modelling of CTE implicates a reduction in anagen duration variance in the pathogenesis ^6^ and not due to an overall reduction in anagen duration, which is a feature of AGA ^7^. The natural history is for continued hair shedding over many years. There may be some seasonal variation in the intensity of hair shedding ^8^. Long-term follow up studies of women with primary CTE ^9^ and histomorphometric and immunohistochemical examination of scalp biopsies in patients with both female pattern hair loss (FPHL) and CTE have confirmed that primary CTE is not a prodrome to AGA ^10^. Nevertheless some women with longstanding CTE will develop con-incidental AGA as they age ^11^. 

**Figure 2.**
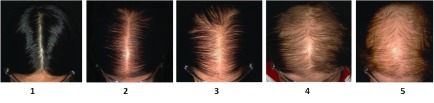
Sinclair Scale for assessment of mid-frontal scalp hair density ^17^.

Primary CTE most commonly occurs suddenly in females between 30 and 50 years of age. Additional clinical features commonly seen in primary CTE include bi-temporal recession of the anterior hairline, a reduction in the thickness of their ponytail diameter ^4^ and trichodynia ^13^. Widening of the central part line suggests AGA, and is not a feature of primary CTE ( Figure 2). 

Other than identification and treatment of a triggering event, such as hypothyroidism, there is no known treatment for acute or chronic TE ^14^. Treatments commonly used for AGA, such as finasteride, cyproterone acetate, spironolactone and flutamide, do not work in TE ^5^. 

Topical minoxidil has been used for over 30 years to treat a variety of hair loss conditions, including AGA. For some years in our clinic we have also used minoxidil orally to treat men and women with androgenetic hair loss, who are either intolerant to topical minoxidil or do not like the look or feel or minoxidil in their hair ^15^. As Minoxidil is only available in Australia as a 10 mg tablet (Loniten), we compounded minoxidil extemporaneously in various doses ranging from 0.25mg to 2.5mg. More recently, a case report demonstrated that low dose oral minoxidil was useful in treating a female with chemotherapy-induced alopecia with oral minoxidil ^16^. There have been no reports or studies that examine the use of oral minoxidil in CTE. Our understanding of the pathogenesis of CTE and the mechanism of action of minoxidil on hair growth suggest it should work in CTE patients, and we have found that women with AGA who are unresponsive to topical minoxidil often respond to oral minoxidil at our clinic. This retrospective study examines the use of low-dose oral minoxidil in women diagnosed with CTE.

## Methods

Examination of patient records for this retrospective chart review was conducted at Sinclair Dermatology practice in Melbourne, Australia. As this was a retrospective review of patient charts, it did not require prior approval from our local institutional ethics committee. The data are the property of Sinclair Dermatology and access was approved by the company. Records between Jan 2012 and Oct 2015 were extracted. Inclusion criteria included female patients with a hair shedding score (HSS) ^12^ of 4–6 without visible mid frontal scalp hair loss (Sinclair stage 1) ^17^ and no hair follicle miniaturization on scalp biopsy. Patients were excluded if they were using other treatments for hair loss whilst taking oral minoxidil. Data pertaining to dosage of oral minoxidil, previous treatments, including topical minoxidil use, blood pressure, side effects, hypertrichosis and trichodynia were extracted from the records. Patient subjective responses were based on a HSS score ( Figure 1) and were recorded at each consultation. The HSS scores prior to starting oral minoxidil, and scores at 6 and 12 months were extracted for analysis. 

Data were analysed using Matlab R2014b statistical software. Difference in HSS at baseline, 6 and 12 months were analysed using the Wilcoxon rank sum test for pair-wise comparisons. Differences in blood pressure at baseline and 6 months were also analysed using Wilcoxon rank sum test. Relationship between outcomes (HSS score at 6 and 12 months) and individual patient specific variables, including age, topical minoxidil, duration of disease, dosage of minoxidil and HSS score at baseline were analysed using a generalised linear regression model.

## Results

**Figure 3.**
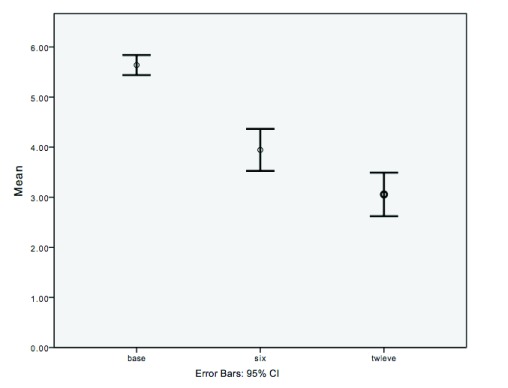
Mean hair shedding scale scores at baseline, six and twelve months.

Thirty-six patients with CTE, who were prescribed oral minoxidil, were included in this analysis. The mean age was 46.9 years (range 20–83 years) and the dosage of oral minoxidil used varied between 0.25 and 2.5 mg with most patients being administered 1 mg. Mean baseline HSS was 5.64. Mean HSS scores improved at 6 and 12 months at 3.9 and 3.05, respectively ( Figure 3). There was a reduction in mean HSS scores from baseline to 6 months of 1.7 (p<0.001) and a reduction in mean HSS scores from baseline to 12 months of 2.58 (p<0.001). Similarly, a mean reduction of 0.89 in HSS scores was noted between 6 months and 12 months (p =0.003). Correlation between the duration of disease and previous topical minoxidil with HSS scores at 6 months (R ^2^ < 0.22) and 12 months (R ^2^ < 0.11) were weak. Eleven patients had previously used 5% topical minoxidil. Of these patients the mean change in the HSS score was higher, although not statistically significant compared to patients who did not use topical minoxidil: mean reductions in HSS score for patients who had previously used and not used topical minoxidil were 2 and 1.56 (p= 0.22) at six months and 3.18 and 2.32 at 12 months (p=0.11). The HSS score improved in 31 patients after 6 months; in 4 patients the HSS score remained the same; and in 1 patient the score increased at the 6-month mark before improving, compared to baseline, at the 12-month mark. After 12 months the HSS score remained equal or improved from baseline in all but 3 patients. 

There were no significant differences between blood pressure at baseline and 6 months (p>0.05). The lowest blood pressure recorded was 90/70. All other patients in the study had a blood pressure above 100/70. Mean change in blood pressure was minus 0.5 mmHg systolic and plus 2.1 mmHg diastolic. Two patients developed transient postural dizziness that resolved with continued treatment. One patient developed ankle oedema. Fourteen women developed facial hypertrichosis. For 6 women this was mild and did not require treatment; 4 patients waxed their upper lip or forehead; and 3 patients had laser hair removal. No patients developed any blood test abnormality. Five women described trichodynia at baseline and all noted improvement or resolution within 3 months. 

Raw data collected during the study Click here for additional data file. Copyright: © 2017 Perera E and Sinclair R 2017 Data associated with the article are available under the terms of the Creative Commons Zero "No rights reserved" data waiver (CC0 1.0 Public domain dedication).

## Discussion

CTE is the persistent shedding of telogen hairs diffusely from the scalp, which is associated with bitemporal recession without widening of the central part. CTE was described as a primary idiopathic disease entity by Whiting in 1996 ^4^. The prognosis for women with CTE is uncertain and women do not go bald ^8^. Rather, the disease has a fluctuating course and the condition may continue for many years. The goal of this retrospective study were to review the use of oral minoxidil in CTE with respect to the response in HSS score and safety.

Minoxidil is a piperidinopyrimidine derivative and a potent arteriolar vasodilator ^5^. Studies examining oral minoxidil demonstrated more rapid and extensive hair growth, compared to topical treatment, in patients with alopecia areata ^12^. A recent study examined the use of oral minoxidil in combination with spironolactone in 100 women with FPHL ^15^. The study demonstrated a reduction of hair loss and hair shedding at 6 and 12 months. Dosages of 0.25 mg of oral minoxidil were used in that study.

Use of oral minoxidil for hair loss has not been extensively reported in the literature to date. Currently, the only suggested treatment for CTE is topical Minoxidil, however results are variable and often disappointing. One study demonstrated an improvement in 55.2% of patients studied, using 5% topical minoxidil for men and 5% topical minoxidil with 50 mg of cyproterone acetate for women ^14^. 25.2% of patients had a moderate response to treatment ^14^.

All the patients in this study demonstrated an improvement at either the 6 month or 12 month mark, with 33 patients improved from baseline at the 12 month mark.

## Abbreviations

FPHL: female pattern hair loss, AGA: androgenetic alopecia, CTE: chronic telogen effluvium, HSS: Hair shedding score.

## Data availability

The data referenced by this article are under copyright with the following copyright statement: Copyright: © 2017 Perera E and Sinclair R

Data associated with the article are available under the terms of the Creative Commons Zero "No rights reserved" data waiver (CC0 1.0 Public domain dedication). 

**Dataset 1: Raw data collected during the study.** DOI, 10.5256/f1000research.11775.d176418 ^18^.
