# Supplementary material for: Treatment of chronic telogen effluvium with oral minoxidil: A retrospective study
Source: F1000Res. 2017 Sep 6;6:1650. [Version 1] doi: 10.12688/f1000research.11775.1 (PMC5676194; doi:10.12688/f1000research.11775.1)
Supplement: Peer review history

# Peer review: Referee response for version 1

**DOI:** 10.5256/f1000research.12721.r26886

**Published:** 2017-11-06

**Authors:** Alex J. Chamberlain

**Affiliations:** 1 Alfred Health, Melbourne, Vic, Australia

This is a useful study, limited somewhat by the retrospective nature and patient numbers. CTE is a challenging condition to manage and this offers another valuable and seemingly safe treatment option.

 I would be interested to know if the authors have a dose preference given their experience for treating CTE. Do they wish to speculate on the mechanism of action of oral minoxidil in hair loss?

 It may be nice to see some of the scalp photographs of the treatment subjects.

 Does the visual shedding scale measure DAILY shedding?

 Clearly a larger study with a standardised dose and a control arm would strengthen the recommendation for the treatment.

I have read this submission. I believe that I have an appropriate level of expertise to confirm that it is of an acceptable scientific standard.

# Peer review: Referee response for version 1

**DOI:** 10.5256/f1000research.12721.r25750

**Published:** 2017-09-21

**Authors:** Victoria M.L. Jolliffe

**Affiliations:** 1 Centre for Cell Biology and Cutaneous Research, Blizard Institute, Queen Mary University of London, London, UK

This is an important piece of work which contributes to the management of an one challenging but common clinical scenario and additional therapeutic interventions which may be of benefit are to be applauded.

 The study is well designed and results robust. There are a couple of further issues which I would like to see addressed or clarified in the text.

1. Address the limitations of a retrospective review.
2. Did the patients have blood work up to exclude any coincidental compounding factors causing effluvium e.g. thyroid/ vit D deficiencies? It would be helpful to know this and indeed see whether is this was the case there was any effect on treatment response in the presence of abnormal blood workup. If they did not have blood work up this should be clearly stated.
3. I assume all the patients included had undergone a scalp biopsy.
4. The dose used was not standardised. This should be mentioned again in the discussion/ limitations of this piece of work and it would be good to know whether side effects - or indeed efficacy - were related to the higher doses used or no difference found.

These are small details on an otherwise really valuable piece of work which will be be very valuable to those practising in the Hair Disorders clinical setting.

I have read this submission. I believe that I have an appropriate level of expertise to confirm that it is of an acceptable scientific standard.
